# Supplementary material for: The inhibitory effect of carboxyl-terminated polyamidoamine dendrimers on dentine host-derived matrix metalloproteinases in vitro in an etch-and-rinse adhesive system
Source: R Soc Open Sci. 2019 Oct 2;6(10):182104. doi: 10.1098/rsos.182104 (PMC6837191; doi:10.1098/rsos.182104)
Supplement: Raw datum (doc. version) [file rsos182104supp1.docx]

Supplementary Material for

**The inhibitory effect of carboxyl-terminated polyamidoamine dendrimers on dentine host-derived matrix metalloproteinases in vitro in an etch-and-rinse adhesive system**

This file includes raw datum used for making figures (Figure 1, 2, 5, 7, 10 and 11). In addition, the file contains raw datum for measuring the intensity ratio of the stretching vibration at ~1660 cm^-1^ to ~3300 cm^-1^ in FTIR, elastic modulus of demineralized dentine and degree of conversion.

**Table and Legends**

 **Table 1**: The percent inhibition of rhMMP9 by different concentrations of PAMAM-COOH, which were used for plotting the Figure1.

**Table 2**: The relative fluorescence intensities of the hybrid layers in the 4 subgroups. The datum were applied to make Figure 2. H_2_O 1d, demineralized dentine interface was pre-treated by deionized water and stored in deionized water for 1day; H_2_O 1y, demineralized dentine interface was pre-treated by deionized water and was subjected to 10,000 thermal cycles. PAM 1d, demineralized dentine interface was conditioned with PAMAM-COOH and stored in deionized water for 1day; PAM 1y, demineralized dentine interface was pre-treated with PAMAM-COOH and was subjected to 10,000 thermal cycles.

**Table 3**: The concentration of ICTP released from demineralized dentine in 4 subgroups, which were used for making Figure 5. Group11, incubated in deionized water without PAMAM-COOH; Group 12, incubated in deionized water with PAMAM-COOH; Group 21, incubated in artificial saliva without PAMAM-COOH; Group 22, incubated in artificial saliva with PAMAM-COOH.

**Table 4**: The intensity ratio of the stretching vibration at ~1660 cm^-1^ to ~3300 cm^-1^ in FTIR. Group 1, demineralized dentine powder; Group 2 demineralized dentine powder treated by PAMAM-COOH.

**Table 5**: The amount of G4-PAMAM-COOH bound to the demineralized dentine powder after adsorption and desorption with deionized water or NaCl. Figure 7 was based on the datum above.

**Table 6**: The elastic modulus (E) of the demineralized dentine discs, measured by atomic force microscope. Group 1, demineralized dentine discs immersed in deionized water; Group 2, demineralized dentine discs immersed in 8 mg/mL G4-PAMAM-COOH.

**Table 7**: Degree of conversion (DC). Group 1, demineralized dentine interface treated by deionized water; Group 2, demineralized dentine interface treated by PAMAM-COOH.

**Table 8**: The relative percentage of resin adhesive permeation in the 4 subgroups. 0 cm, 0 cm of H_2_O pressure; 5 cm, 5 cm of H_2_O pressure; Water, demineralized dentin interface pre-treated with deionized water; PAMAM, demineralized dentine interface conditioned with PAMAM-COOH. The datum were used for graphing Figure 10.

**Table 9**: The value of ultimate tensile strength (UTS) in the 4 subgroups. The datum were applied to make Figure 11. H_2_O 1d, demineralized dentine interface was pre-treated by deionized water and stored in deionized water for 1day; H_2_O 1y, demineralized dentine interface was pre-treated by deionized water and was subjected to 10,000 thermal cycles. PAMAM 1d, demineralized dentin interface was conditioned with PAMAM-COOH and stored in deionized water for 1day; PAMAM 1y, demineralized dentine interface was pre-treated with PAMAM-COOH and was subjected to 10,000 thermal cycles.
